# Frequency Distribution of Neoplastic and Non-neoplastic Thyroid Lesions in Relation to Patient Age and Gender

**DOI:** 10.7759/cureus.98128

**Published:** 2025-11-30

**Authors:** Zainab Jamil, Safana Sadaf, Saima Batool, Sarosh Iqbal, Mehroosh Shakeel, Sania Shuja

**Affiliations:** 1 Histopathology, Fatima Memorial Hospital College of Medicine and Dentistry, Lahore, PAK; 2 Histopathology, Shaukat Khanum Memorial Cancer Hospital Lahore, Lahore, PAK; 3 Histopathology, Al-Aleem Medical College, Gulab Devi Hospital, Lahore, PAK; 4 Pathology, Shalamar Medical and Dental College, Lahore, Lahore, PAK

**Keywords:** age, gender, multinodular goiter, neoplastic, non-neoplastic, papillary carcinoma, thyroid lessions

## Abstract

Background: Thyroid lesions, ranging from benign nodules to malignant neoplasms, are among the most common endocrine pathologies. Understanding local histopathologic patterns is essential for guiding clinical management, surgical planning, and resource allocation.

Objective: To assess the frequency and distribution of neoplastic and non-neoplastic thyroid lesions in relation to patient age and gender at a tertiary care center in Lahore, Pakistan.

Methods: This retrospective study analyzed 305 thyroidectomy specimens collected over five years (2018-2022) at Shalamar Hospital, Lahore. Patients aged 10-89 years who underwent lobectomy, subtotal, or total thyroidectomy were included. Data on age, gender, and histopathologic classification were extracted from departmental records. Data were analyzed using SPSS version 25 (IBM Corp., Armonk, NY, USA).

Results: Among 305 patients, 244 (80.0%) were female and 61 (20.0%) male, with a mean age of 44.0 ± 15.2 years. Neoplastic lesions comprised 168 cases (55.1%), with benign follicular adenoma accounting for 20.0% and malignant neoplasms for 35.1%, predominantly papillary carcinoma (24.9%). Non-neoplastic lesions accounted for 137 cases (44.9%), most commonly multinodular goiter (24.9%). Neoplastic lesions were significantly more frequent in females (59.8%) than males (36.1%) (χ² = 11.42, p = 0.001), and patients with neoplastic lesions were slightly older than those with non-neoplastic disease (45.6 ± 14.5 vs 42.0 ± 15.9 years, p = 0.041).

Conclusion: Neoplastic lesions slightly outnumbered non-neoplastic lesions, with papillary carcinoma and multinodular goiter being the most common subtypes, respectively. Age and gender influenced lesion distribution, highlighting their importance in preoperative counseling and surgical planning.

## Introduction

The thyroid gland is a midline endocrine organ located in the anterior cervical compartment, positioned inferior to the thyroid cartilage of the larynx and typically extending from C5 to T1 vertebral levels. Anatomically, it consists of right and left lobes interconnected by a thin isthmus. Functionally, the gland is integral to systemic homeostasis, synthesizing thyroxine (T4) and triiodothyronine (T3) from its follicular epithelium and calcitonin from parafollicular (C) cells, thereby modulating metabolic activity, somatic growth, and calcium homeostasis [1,2]. Thyroid lesions - ranging from benign nodular disorders to various neoplasms - represent one of the most frequent endocrinological pathologies worldwide.

Globally, clinically palpable thyroid nodules affect 4-7% of the adult population, while autopsy and imaging studies suggest up to 25% prevalence [3]. Only a small fraction (~5%) of detected nodules prove malignant, and most thyroid swelling cases are benign [4]. Studies have also indicated that the age‑standardized thyroid cancer incidence of about 10.1 per 100,000 women versus 3.1 per 100,000 men globally. Despite regional disparities, the incidence was roughly five times higher in high Human Development Index (HDI) countries compared to low or middle. Mortality remained low (<1 per 100,000) in both sexes, suggesting a substantial overdiagnosis effect from heightened screening practices [5].

This pattern is mirrored in regional South Asian data. In Peshawar (2024, Khyber Journal Study), a cross‑sectional thyroidectomy cohort of 100 patients (85 females, 15 males) revealed that non‑neoplastic lesions were predominant among women aged 30-50, whereas males, particularly younger men, exhibited a proportionally higher incidence of neoplastic - including malignant - lesions, highlighting a clear sex‑ and age‑related disparity in histopathological distribution [6]. A large retrospective series from tertiary hospitals in Lahore (2017-2021) reviewed 1,217 surgical specimens and identified 333 thyroid lesions, of which approximately 91% were neoplastic. Multinodular and adenomatous colloid goiters remained the most frequent lesions, while papillary carcinoma (10.2%) and follicular adenoma (6.6%) were the leading neoplastic subtypes. Interestingly, overall lesion frequency was higher in males (56.6%), and age‑related distribution showed statistically significant variation (p < 0.001), reinforcing both regional and global epidemiologic trends that thyroid disease burden is strongly influenced by age and sex stratification [7]. 

Thyroid lesion epidemiology shows clear sex and age dependence, with women, due to estrogen influence and autoimmune predisposition, accounting for up to four to seven times more benign nodules, while males carry a higher relative malignancy risk, especially at <30 or >60 years [8,9]. Age‑related risk rises with iodine deficiency, low‑level radiation, metabolic dysregulation, and autoimmune thyroiditis. Overdiagnosis in high‑HDI settings from ultrasound and fine needle aspiration cytology (FNAC) has increased detection of indolent neoplasms, whereas hospital cohorts may overreport neoplastic cases due to referral bias [9]. These trends justify age‑ and sex‑specific risk stratification, highlight the importance of identifying true neoplastic burden, and form the rationale for assessing the frequency of thyroid lesions by age and gender in our setting.

Pakistan’s iodine deficiency shapes the histopathological spectrum of thyroid disease, with benign nodular lesions predominating and neoplastic lesions showing age‑ and gender‑specific patterns. Local data are limited, yet essential for risk stratification, surgical planning, and public health strategies. Shalamar Hospital, Lahore, as a tertiary referral center for both urban and rural patients, offers a representative cohort to analyze the frequency and distribution of neoplastic and non‑neoplastic thyroid lesions across age and gender, providing evidence to guide optimized clinical management in this setting.

## Materials and methods

**Figure 1:**
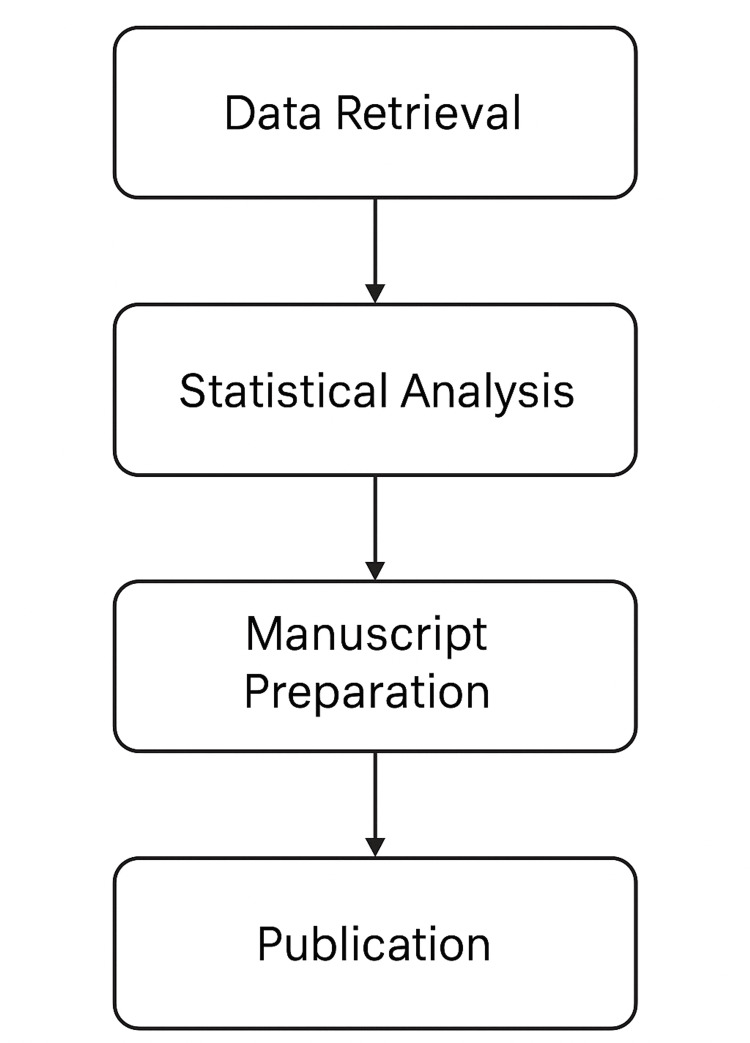
Structured workflow followed in the study

This retrospective study was conducted at Shalamar Hospital, Lahore, over 10 months, analyzing thyroidectomy specimens (lobectomy, subtotal, and total thyroidectomy) from the past five years (2018-2022). Data were collected through a convenience sampling technique. All pathology reports of patients aged 10-89 years who underwent thyroid surgery were included, while congenital thyroid lesions (e.g., thyroglossal or branchial cysts) and secondary malignancies (e.g., lymphoma or metastatic tumors from lung, renal, or adrenal primaries) were excluded. A convenience sampling technique was employed, and data were extracted retrospectively from the histopathology records of the pathology department following institutional approval and IRB clearance (approval SMDC-IRB/AL/30/2023). A self‑designed proforma was used to collect variables, including age, gender, and histopathological classification into neoplastic and non‑neoplastic lesions with subcategories. Data were analyzed using SPSS version 25 (IBM Corp., Armonk, NY, USA), with descriptive statistics including frequency, percentage, mean, and standard deviation (SD) applied to assess the age‑ and gender‑wise distribution of thyroid lesions. The study followed a structured workflow involving data retrieval, statistical analysis, and manuscript preparation for publication (Figure 1).

## Results

**Figure 2:**
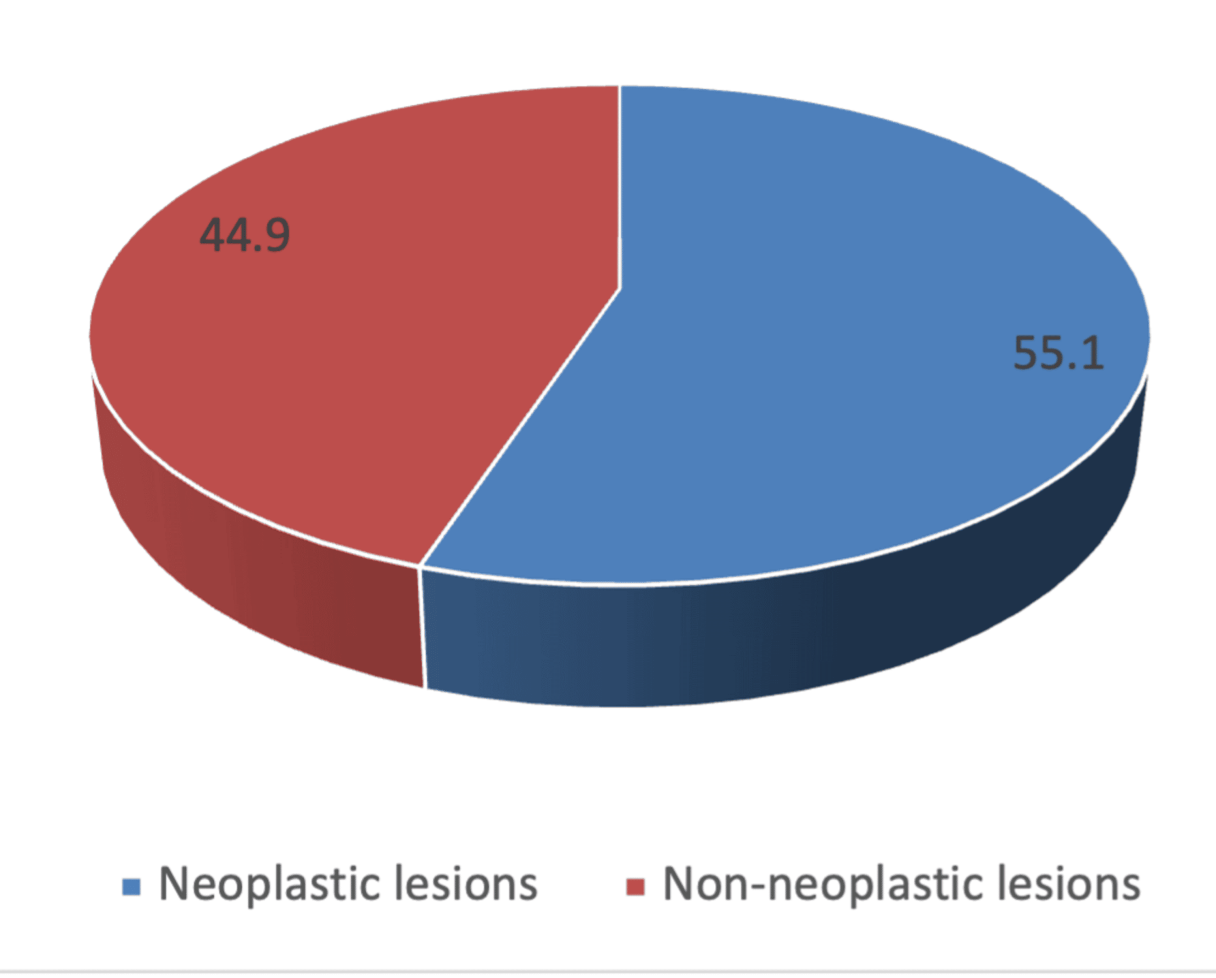
Overall distribution of thyroid lesions among 305 thyroidectomy specimens, showing neoplastic lesions (55.1%) and non-neoplastic lesions (44.9%). This figure graphically displays the proportion of neoplastic (55.1%) and non-neoplastic (44.9%) thyroid lesions identified in the study. It provides a visual overview of the overall lesion pattern among all cases analyzed.

A total of 305 thyroidectomy specimens were analyzed in this study. The majority of patients were female (n = 244, 80.0%), while male patients comprised 20.0% (n = 61). The mean age of the study population was 44.0 ± 15.2 years, with the highest proportion of patients falling within the 30-39 years (27.2%) and 40-49 years (24.3%) age groups, followed by 50-59 years (18.0%). Only a small proportion of cases were observed in the younger (10-19 years, 3.3%) and older (70-89 years, 4.3%) age categories (Table 1, Figure 2).

**Table 1:** Baseline demographic characteristics and age group distribution of patients with thyroid lesions (n = 305)

Variable	Category	n (%) / Mean ± SD
Gender	Female	244 (80.0)
	Male	61 (20.0)
Age (years)	Mean ± SD	44.0 ± 15.2
Age Group (years)	10–19	10 (3.3)
	20–29	43 (14.1)
	30–39	83 (27.2)
	40–49	74 (24.3)
	50–59	55 (18.0)
	60–69	27 (8.9)
	70–89	13 (4.3)
Total Cases	—	305 (100.0)

Out of 305 thyroid lesions, 168 cases (55.1%) were neoplastic, while 137 cases (44.9%) were non-neoplastic (Figure 2). Among the neoplastic lesions, benign neoplasms accounted for 61 cases (20.0%), all of which were follicular adenomas. The malignant neoplasms comprised 107 cases (35.1%), of which papillary carcinoma was the most frequent subtype (n = 76, 24.9%), followed by follicular carcinoma (n = 12, 3.9%), medullary carcinoma (n = 9, 3.0%), anaplastic carcinoma (n = 6, 2.0%), and a few cases of other malignancies (n = 4, 1.3%).

In the non-neoplastic group (n = 137, 44.9%), the most common lesion was multinodular goiter (n = 76, 24.9%), followed by Hashimoto thyroiditis (n = 27, 8.9%), colloid nodule (n = 18, 5.9%), Graves’ disease (n = 7, 2.3%), thyroid cysts (n = 5, 1.6%), and subacute thyroiditis (n = 4, 1.3%) (Table 2).

**Table 2:** Frequency distribution of neoplastic and non-neoplastic thyroid lesions with subcategories (n = 305). It presents the results of the chi-square test showing a statistically significant gender difference in lesion type distribution (p = 0.001).

Lesion category	n	% (of total)
Neoplastic (total)	168	55.1%
Benign neoplasm — Follicular adenoma	61	20.0%
Malignant neoplasms (total)	107	35.1%
Papillary carcinoma	76	24.9%
Follicular carcinoma	12	3.9%
Medullary carcinoma	9	3.0%
Anaplastic carcinoma	6	2.0%
Other malignant	4	1.3%
Non-neoplastic (total)	137	44.9%
Multinodular goiter	76	24.9%
Hashimoto thyroiditis	27	8.9%
Colloid nodule	18	5.9%
Graves’ disease	7	2.3%
Thyroid cyst	5	1.6%
Subacute thyroiditis	4	1.3%

When analyzed by gender, neoplastic lesions were significantly more common in females (146 cases, 59.8%) compared to males (22 cases, 36.1%), whereas non-neoplastic lesions were more frequent in males (39 cases, 63.9%) compared to females (98 cases, 40.2%). This difference was statistically significant (χ² = 11.42, df = 1, p = 0.001) (Table 3).

**Table 3:** Comparison of neoplastic and non-neoplastic thyroid lesions by gender with Chi-square analysis (n = 305)

Category	Female n (%)	Male n (%)	Total n (%)	χ² (df=1)	p-value
Neoplastic	146 (59.8%)	22 (36.1%)	168 (55.1%)	11.42	0.001
Non-neoplastic	98 (40.2%)	39 (63.9%)	137 (44.9%)		
Overall	244 (80.0%)	61 (20.0%)	305 (100.0%)		

Age comparison between groups showed that patients with neoplastic lesions had a slightly higher mean age (45.6 ± 14.5 years) than those with non-neoplastic lesions (42.0 ± 15.9 years). This difference was statistically significant (t = 2.05, df = 303, p = 0.041) (Table 4).

**Table 4:** Comparison of mean age between neoplastic and non-neoplastic thyroid lesions with independent samples t-test (n = 305). The independent samples t-test indicates that patients with neoplastic lesions were significantly older on average than those with non-neoplastic lesions (p = 0.041).

Lesion group	Mean age (years) ± SD	t-test (df = 303)	p-value
Neoplastic (n=168)	45.6 ± 14.5	2.05	0.041
Non-neoplastic (n=137)	42.0 ± 15.9		
Overall (n=305)	44.0 ± 15.2		

## Discussion

This retrospective study of 305 thyroidectomy specimens from Shalamar Hospital, Lahore, revealed that neoplastic lesions comprised 55.1% of cases, while non-neoplastic lesions accounted for 44.9%. Among the neoplastic lesions, papillary thyroid carcinoma (PTC) was the most prevalent, constituting 24.9% of all cases. Multinodular goiter (MNG) was the most common non-neoplastic lesion, representing 24.9% of cases. The study also observed a significant female predominance (80.0%) and a slightly higher mean age in patients with neoplastic lesions (45.6 years) compared to those with non-neoplastic lesions (42.0 years). The predominance of neoplastic lesions, particularly PTC, aligns with findings from other regional studies. For instance, a study by Santosh et al. (2023) reported that PTC was the most common malignant thyroid lesion in their cohort [10]. Similarly, Chandanwale et al. (2022) observed that thyroidectomy for both neoplastic and non-neoplastic lesions was most commonly performed in the third and fourth decades of life, with a higher incidence in females. These findings underscore the consistency of our results with broader regional trends [11]. The higher mean age observed in patients with neoplastic lesions is consistent with global patterns, where differentiated thyroid cancers, such as PTC, are more commonly diagnosed in older adults. However, it's noteworthy that PTC can also occur in younger individuals, as highlighted by Xu et al. (2023), who reported an increase in PTC incidence among individuals aged 30-34 years [12]. Moreover, comparisons with regional data show both similarities and expected variability. Several recent Pakistani and South Asian institutional series report MNG and benign nodular disease as frequent indications for thyroidectomy, with PTC remaining the leading malignancy when cancers are present. For example, multi-center and single-center studies from Pakistan reported high proportions of MNG among non-neoplastic lesions and papillary carcinoma as the dominant malignant subtype, underlining consistent regional pathology patterns [13]. These findings support the external validity of our results for tertiary care practice in South Asia [14]. Similarly, the strong female predominance observed in our cohort (female: male ≈ 4:1) is consistent with the well-established sex disparity in thyroid disease. Thyroid nodular disease and differentiated thyroid cancers are more frequently detected in women-often attributed to hormonal influences, autoimmune predisposition, and healthcare-seeking behaviors-though some population studies suggest that the true subclinical prevalence may be more similar across sexes [15]. A study by Naz et al. (2024) highlighted a significant gender disparity in thyroid lesions, with non-neoplastic conditions more common in females, particularly aged 30 to 50 [6]. In contrast, males had a slightly higher occurrence of neoplastic lesions, including malignancies in younger individuals. This suggests that gender-specific strategies may be beneficial in managing thyroid diseases.

This study provides up-to-date data on the types of thyroid lesions seen in surgical specimens over five years. The findings help guide preoperative counseling, surgical planning, and diagnostic decisions - such as estimating cancer risk from FNA results, deciding the extent of surgery, and identifying cases needing genetic counseling. They also support improving diagnostic pathways, like standardizing ultrasound reports and applying molecular tests, to avoid unnecessary surgery while ensuring malignancies are managed appropriately. Being a single-center, retrospective study, our results reflect only surgically treated patients and may overrepresent symptomatic or suspicious nodules. We lacked molecular testing and detailed clinical data like nodule size, imaging features, and follow-up outcomes, which limits broader generalization. Future research should collect prospective, multi-center data with molecular testing and long-term outcomes. Standardized risk assessment tools and targeted molecular panels could help identify high-risk nodules while minimizing unnecessary thyroidectomies.

## Conclusions

In this five-year institutional study of 305 thyroidectomy specimens, neoplastic lesions accounted for a slight majority, with papillary thyroid carcinoma being the most common malignancy, while multinodular goiter was the predominant non-neoplastic lesion. Females were disproportionately affected, and patients with neoplastic lesions were slightly older than those with non-neoplastic disease. These findings provide valuable insights for preoperative counseling, surgical planning, and resource allocation, and highlight the importance of age and gender in thyroid lesion presentation. Future prospective, multi-center studies incorporating molecular testing and long-term outcomes are warranted to further refine risk stratification and optimize patient management.

## Appendices

Data collection proforma

Study Title: Frequency Distribution of Neoplastic and Non-Neoplastic Thyroid Lesions in Relation to Patient Age and GenderSetting: Department of Pathology, Shalamar Hospital, LahoreDuration: 2018-2022

VariableResponse / Entry

Patient Information

Study ID / Serial No.______________________________

Hospital Record No.______________________________

Date of Surgery______________________________

Type of Surgery☐ Lobectomy  ☐ Subtotal Thyroidectomy  ☐ Total Thyroidectomy

Age (years)______________________________

Gender☐ Male  ☐ Female

Residential Area☐ Urban  ☐ Rural

Histopathological Findings

Lesion Category☐ Neoplastic  ☐ Non-Neoplastic

If Neoplastic:

Type of Neoplasm☐ Benign (Follicular Adenoma)  ☐ Malignant

Subtype (if Malignant)☐ Papillary Carcinoma  ☐ Follicular Carcinoma  ☐ Medullary Carcinoma  ☐ Anaplastic Carcinoma  ☐ Other (specify): __________

If Non-Neoplastic:

Subtype☐ Multinodular Goiter  ☐ Hashimoto Thyroiditis  ☐ Colloid Nodule  ☐ Graves’ Disease  ☐ Thyroid Cyst  ☐ Subacute Thyroiditis  ☐ Other: __________

Histopathology Report No.______________________________

Microscopic Description Summary__________________________________________________________________________________________

Pathologist’s Initials______________________________

Additional Notes

Special Stains / IHC Used☐ Yes ☐ No  If Yes, specify: ______________________________

Incidental Findings_____________________________________________

Comments_____________________________________________

Data Verification

Verified By______________________________

Date______________________________

Signature______________________________
